# Virtual Versus In-Person Focus Groups: Comparison of Costs, Recruitment, and Participant Logistics

**DOI:** 10.2196/jmir.6980

**Published:** 2017-03-22

**Authors:** Douglas J Rupert, Jon A Poehlman, Jennifer J Hayes, Sarah E Ray, Rebecca R Moultrie

**Affiliations:** ^1^ Center for Communication Science RTI International Research Triangle Park, NC United States; ^2^ Center for Communication Science RTI International Atlanta, GA United States

**Keywords:** focus groups, virtual systems, online systems, videoconferencing, qualitative research, communication, mobile apps, diabetes mellitus

## Abstract

**Background:**

Virtual focus groups—such as online chat and video groups—are increasingly promoted as qualitative research tools. Theoretically, virtual groups offer several advantages, including lower cost, faster recruitment, greater geographic diversity, enrollment of hard-to-reach populations, and reduced participant burden. However, no study has compared virtual and in-person focus groups on these metrics.

**Objective:**

To rigorously compare virtual and in-person focus groups on cost, recruitment, and participant logistics. We examined 3 focus group modes and instituted experimental controls to ensure a fair comparison.

**Methods:**

We conducted 6 1-hour focus groups in August 2014 using in-person (n=2), live chat (n=2), and video (n=2) modes with individuals who had type 2 diabetes (n=48 enrolled, n=39 completed). In planning groups, we solicited bids from 6 virtual platform vendors and 4 recruitment firms. We then selected 1 platform or facility per mode and a single recruitment firm across all modes. To minimize bias, the recruitment firm employed different recruiters by mode who were blinded to recruitment efforts for other modes. We tracked enrollment during a 2-week period. A single moderator conducted all groups using the same guide, which addressed the use of technology to communicate with health care providers. We conducted the groups at the same times of day on Monday to Wednesday during a single week. At the end of each group, participants completed a short survey.

**Results:**

Virtual focus groups offered minimal cost savings compared with in-person groups (US $2000 per chat group vs US $2576 per in-person group vs US $2,750 per video group). Although virtual groups did not incur travel costs, they often had higher management fees and miscellaneous expenses (eg, participant webcams). Recruitment timing did not differ by mode, but show rates were higher for in-person groups (94% [15/16] in-person vs 81% [13/16] video vs 69% [11/16] chat). Virtual group participants were more geographically diverse (but with significant clustering around major metropolitan areas) and more likely to be non-white, less educated, and less healthy. Internet usage was higher among virtual group participants, yet virtual groups still reached light Internet users. In terms of burden, chat groups were easiest to join and required the least preparation (chat = 13 minutes, video = 40 minutes, in-person = 78 minutes). Virtual group participants joined using laptop or desktop computers, and most virtual participants (82% [9/11] chat vs 62% [8/13] video) reported having no other people in their immediate vicinity.

**Conclusions:**

Virtual focus groups offer potential advantages for participant diversity and reaching less healthy populations. However, virtual groups do not appear to cost less or recruit participants faster than in-person groups. Further research on virtual group data quality and group dynamics is needed to fully understand their advantages and limitations.

## Introduction

### Overview

Qualitative research is a critical component of public health interventions and evaluations, providing in-depth information that can be difficult to obtain through surveys and other quantitative methods [1]. Focus groups, in particular, are a valuable tool for identifying and dissecting the knowledge, attitudes, and perceptions that influence individuals’ behavior as well as the barriers and facilitators to behavioral change [2,3]. Market research firms are increasingly offering and promoting the use of virtual focus groups to collect qualitative data. Virtual groups are generally defined as qualitative research sessions in which multiple individuals congregate remotely to discuss a specific topic [4,5]. Virtual focus groups may be conducted via phone, chat, or video platforms and may be held either synchronously during a 1- to 2-hour period or asynchronously over multiple days [5].

### Presumed Benefits of Virtual Focus Groups

Hypothetically, virtual focus groups offer multiple advantages over traditional, in-person focus groups [5-7]. First, virtual groups theoretically should be less expensive, eliminating travel costs for research staff as well as other incidental expenses (eg, snacks, parking fees). Second, the turnaround time for virtual groups should be faster because they eliminate travel between research sites and, depending on the platform, offer instantaneous transcripts. Third, virtual sessions should facilitate greater geographic diversity both within and across focus groups by enrolling participants from a greater number of locations.

Fourth, virtual groups should reach populations that are often excluded from or under-represented in traditional focus groups, including rural residents, individuals with less than a high school education, individuals of lower socioeconomic status, and individuals with health and mobility impairments. Likewise, researchers can theoretically use virtual platforms to convene individuals from rare populations (eg, low prevalence health conditions) where in-person gatherings would be impossible. Finally, virtual focus groups should reduce the travel and logistical burdens on participants, resulting in higher show rates and faster recruitment.

### Evidence on Virtual Focus Groups

Despite the promise of this methodology, the evidence base for virtual focus groups is extremely thin. Few studies have examined whether these hypothesized benefits materialize in practice, and even fewer studies have rigorously compared traditional and virtual focus groups on the aforementioned metrics [6,8]. In terms of cost, no study has directly compared the expenses of virtual and in-person focus groups, although several articles report anecdotal cases of unquantified cost savings [5,7-10]. Likewise, only 1 study has directly examined recruitment differences between traditional and chat focus groups, finding that virtual group participants were slightly younger than in-person participants [11]. Finally, no study has compared the participant logistics of traditional and virtual focus groups, such as travel and preparation time, technology requirements, interference from nearby individuals, and barriers to participation.

In addition to the lack of direct comparisons between traditional and virtual groups, the few studies that have examined virtual focus groups have several methodological limitations. First, the modes compared in the literature are very limited. Most of the chat-based groups have been asynchronous rather than real time [7,8,12-14], and only 1 study has examined video-based groups [15], which are an increasingly common offering from market research firms. Second, almost all virtual group participants either have been drawn from panels of known Internet users [14,16] or represent specialized populations, such as college students and active military personnel [6-9,13,15,17], all of which restrict the generalizability of study findings.

Third, many studies employ weak or subjective measurements to assess virtual focus group characteristics, including expert rankings, research team commentaries, or self-reported participant preferences [4,7,9,14,16,18]. Almost no study has employed rigorous or objective measures to assess virtual group performance [15]. Finally, most studies that compare focus group modes lack strong experimental controls, with studies frequently employing different moderators, durations, sample sizes, and eligibility criteria by mode [8,16] or failing to employ any type of comparison group [7,9,13]. Ultimately, these limitations dilute the evidence base on virtual focus groups, making it difficult to assess their true benefits and limitations.

The purpose of this study was to address these gaps in evidence by rigorously comparing virtual and traditional focus groups on cost, recruitment, and participant logistics. We examined 3 focus group modes—in-person, live chat, and video—and instituted strong experimental controls to ensure a fair comparison. Specifically, we sought to answer the following 3 research questions:

1. Cost: How do costs—both projected and actual—differ by focus group mode?

2. Recruitment: How do recruitment timing and participant characteristics differ by focus group mode?

3. Participant Logistics: How do participant logistics—such as difficulty attending and preparation time—differ by focus group mode?

## Methods

### Study Design

**Figure 1:**
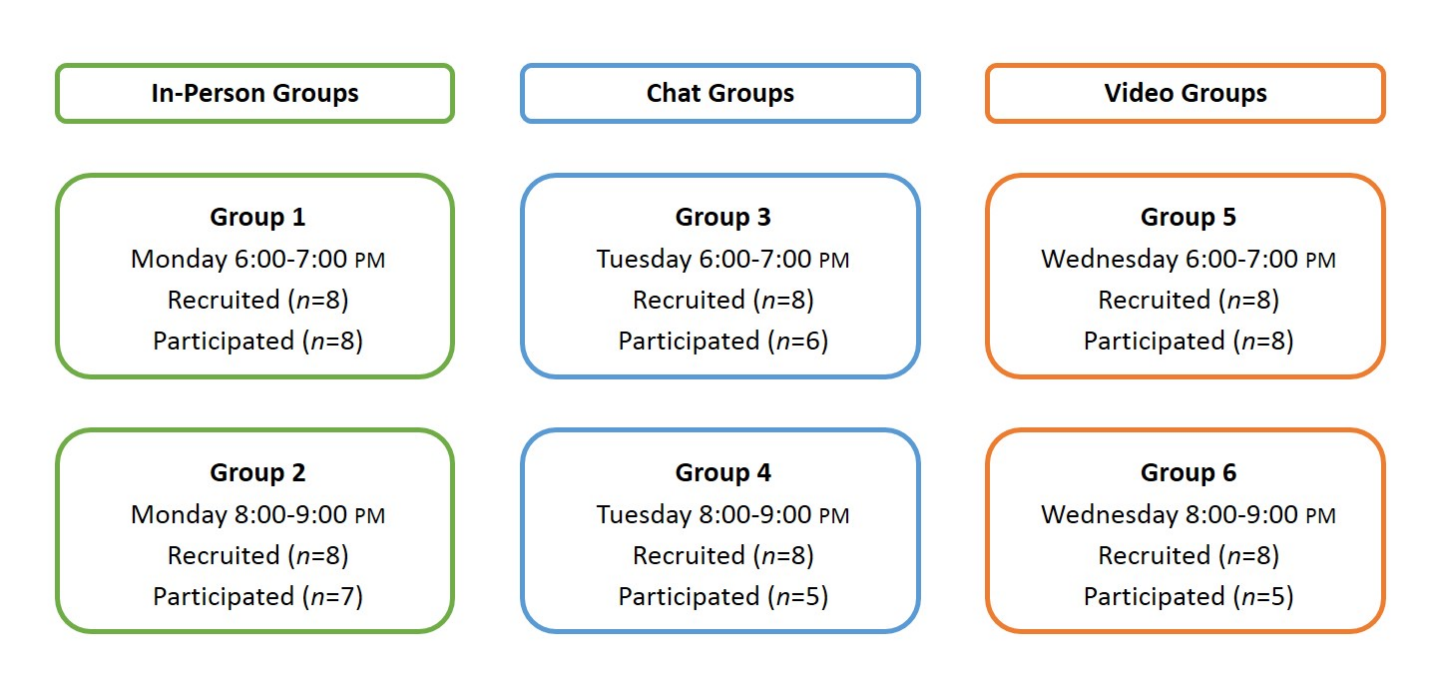
Study design and sample sizes.

We planned and conducted a series of 6 focus groups using 3 different modes—in-person (n=2), live chat (n=2), and video (n=2)—with individuals who had type 2 diabetes (Figure 1). The topic of all 6 groups was using technology to communicate with health care providers, with groups discussing actual and intended use of email, patient portals, and wearable devices to share health information with clinicians. We selected this topic because it was applicable across all demographic groups (eg, sex, age, race, education, income), and we structured the moderator guide so that participants could engage in group discussion regardless of their actual technology use.

We conducted the groups in August 2014 at the same times of day (6:00-7:00 PM and 8:00-9:00 PM Eastern Daylight Time) from Monday to Wednesday during a single week. We held the in-person groups in Atlanta, GA. Each focus group comprised a 1-hour moderated discussion, and we recruited 8 people per group with no over-recruitment. We ultimately enrolled 48 individuals in the study, of which 39 individuals participated in the groups.

### Study Population, Eligibility Criteria, and Participant Quotas

The study population comprised individuals diagnosed with type 2 diabetes. We selected this population because public health professionals often conduct formative research with a specific illness population and because type 2 diabetes is broadly distributed across demographic groups (ie, study population does not introduce demographic bias).

Within this population, we established eligibility criteria to ensure participants could actively engage in the discussion and were not frequent research participants (Table 1). These criteria mirror the eligibility criteria typically adopted in other formative health research studies [19-21]. For the virtual focus groups, we also required individuals to have sufficient Internet access to participate in an online discussion. Chat group participants needed to have at least a dial-up Internet connection, and video group participants needed to have a high-speed Internet connection.

**Table 1:** Participant eligibility criteria.

Eligibility criteria	Rationale
Age 18 years or older	Ensures participants are adults who can consent to enroll in the study Ensures participants make own health care decisions
Diagnosed with type 2 diabetes	Ensures participants are members of the same illness population
Not employed in health care or research fields	Reduces likelihood of response bias
No focus group participation in last 6 months	Reduces likelihood of response bias
English as primary language	Ensures participants can adequately engage in group discussions
Have a regular health care provider	Ensures focus group topic is relevant to participants
Visited health care provider within the last year	Ensures focus group topic is relevant to participants

In addition to these eligibility criteria, we also set several participant quotas for the recruitment firms (Table 2). We established these quotas both to ensure greater demographic diversity within each focus group and to assess recruitment of key demographic groups by mode (for example, we wanted to examine recruitment of less educated individuals for virtual groups versus in-person groups).

**Table 2:** Participant enrollment quotas.

Category	Enrollment quota per group
Age	Minimum of 2 individuals aged 35 years or younger
	Minimum of 2 individuals aged 55 years or older
Sex	Minimum of 3 males
	Minimum of 3 females
Education	Minimum of 2 individuals with a high school education or less
	Minimum of 2 individuals with some college education or more

### Platform and Vendor Selection

First, we solicited bids from multiple vendors to measure average recruitment and hosting costs by focus group mode. Specifically, we collected itemized bids from 4 recruitment firms to recruit participants for all 3 modes, and we collected itemized bids from these same 4 firms to host the in-person focus groups. We also collected itemized bids from 6 Web-based vendors (3 live chat, 3 video) for hosting the virtual groups. We used these bids to calculate projected costs by mode.

We ultimately selected a single recruitment firm to host the in-person focus groups and recruit participants for all 6 groups across all modes. We selected a single firm to ensure consistent recruitment practices across modes and eliminate the possibility that recruitment strategies would confound mode-versus-mode comparisons. We also selected one online vendor to host the chat focus groups and another online vendor to host the video focus groups.

When selecting vendors, we considered cost competitiveness, past experience, and—in the case of virtual focus groups—platform functionality. Specifically, we required that the virtual group platform offer interactive capabilities, such as electronic consent forms, Web-based exit questionnaires, handout sharing and markup, and polling. Although we did not use all of these features in this study, these capabilities are essential for many scientific and public health research studies and were important to consider. Given that the study discussed personal health information, we also required platforms that could guarantee data privacy and that would protect participants’ identities at a level sufficient to meet or exceed the institutional review board guidelines. Because no publicly available platform (eg, Skype, Facetime) offered these features, we ultimately selected proprietary virtual group platforms to ensure that study results were generalizable to a wide range of scientific research studies.

### Recruitment and Enrollment

We selected a single recruitment firm with national reach to recruit participants for all 3 modes. The firm was blinded to the study’s purpose and was not aware that we would be comparing recruitment by mode. To ensure experimental control, the recruitment firm assigned separate recruiters to each focus group mode and provided them with identical eligibility requirements (with the exception of Internet speed). To minimize bias (eg, mode preference) and learning curves (eg, recruitment skill improves with time), the recruiters were blinded to recruitment efforts for the other modes. Recruiters had 2 weeks to complete enrollment. During the enrollment period, recruiters provided daily spreadsheets showcasing their progress.

For all modes, recruiters identified potential participants using contact databases and advertisements. The recruiters contacted potentially eligible individuals by telephone, screened them for eligibility using a 25-item questionnaire, and scheduled eligible individuals for focus groups at preselected dates and times.

### Data Collection

We developed a semistructured moderator guide containing questions and probes on the topics of email communication with health care providers, patient portals, sensors and wearable devices (eg, Fitbit), automatic sharing of personal biomedical information (eg, blood glucose level) with health care providers, and privacy concerns or preferences related to technology. A single moderator conducted all 5 focus groups using this same guide.

#### In-Person Groups

For the in-person groups, we conducted 1-hour focus groups at a market research facility in Atlanta. We administered written informed consent to participants upon arrival, and a trained moderator conducted each group by asking questions, probing for details, and leading participants in verbal discussion. At the end of each session, participants completed a 15-item hardcopy exit questionnaire. We also audio and video recorded the sessions and produced verbatim transcripts. Participants received a US $75 incentive after each session.

#### Chat Groups

**Figure 2:**
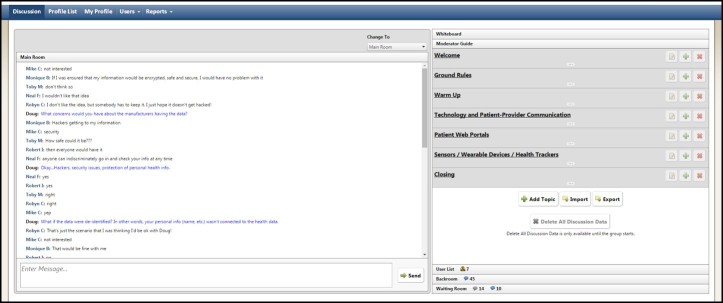
Chat focus group screenshot example.

For the chat groups, we conducted 1-hour focus groups on a real-time live chat platform hosted by an online research vendor (see Figure 2 for example). We had emailed consent forms and confirmation letters with login credentials to participants several days in advance. Upon login, participants acknowledged an electronic consent form. A trained moderator posted questions and probes from the guide in a chat box, and participants typed responses visible to the entire group. At the end of each session, participants completed an 18-item Web-based exit questionnaire. The platform produced verbatim transcripts of the typed responses from the open group discussion. Participants were mailed a US $75 incentive after each session.

#### Video Groups

**Figure 3:**
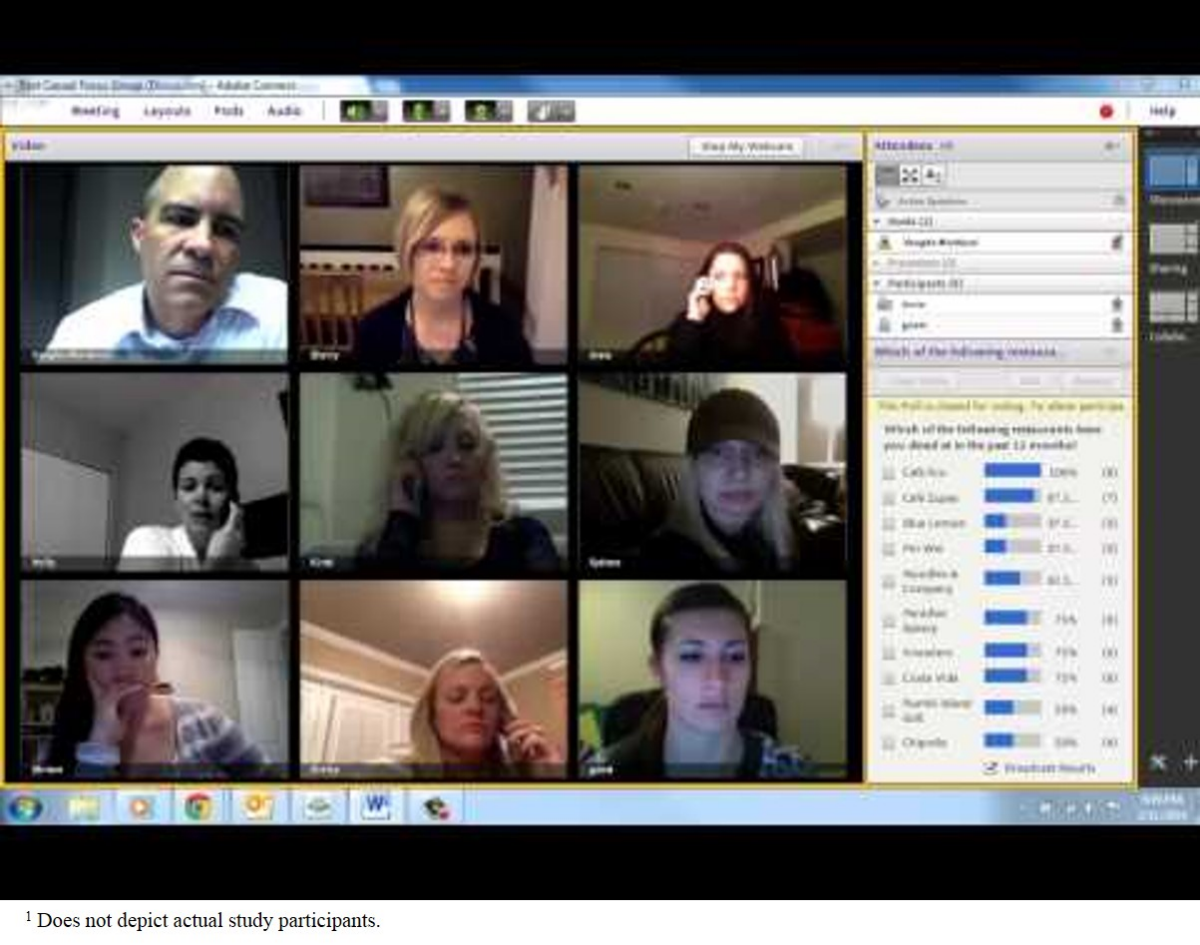
Video focus group screenshot example.

For the video groups, we conducted 1-hour focus groups on a real-time video platform hosted by an online research vendor (see Figure 3 for example). We had emailed consent forms and confirmation letters with login credentials to participants several days in advance. Upon login, participants acknowledged an electronic consent form. Participants joined the sessions using Web cameras connected to their computer or mobile devices. (If participants did not have a Web camera, we supplied them with one.) The moderator and participants were able to see and hear other individuals’ video feeds on screen, and the moderator conducted each group by asking questions, probing for details, and leading participants in verbal discussion. At the end of each session, participants completed an 18-item Web-based exit questionnaire. We also audio and video recorded the sessions and produced verbatim transcripts. Participants were mailed a US $75 incentive after each session.

During all groups, a note taker documented major themes in the discussion. The note taker also completed an observer worksheet that documented participant logistics, such as late arrivals, no-shows, cancellations, and early departures.

### Measurements and Data Analysis

#### Cost Measures

We measured projected costs by averaging the itemized cost estimates on the vendor bids we solicited. Specifically, we measured recruitment costs (eg, recruitment, proposed incentives) by averaging costs for each mode across the 4 recruitment firm bids. We used an identical approach for measuring in-person facility costs (eg, facility rental, video recording, transcripts) and virtual platform costs (eg, platform rental, management fees, recording, transcripts). We measured projected travel expenses by securing an estimate through our institutional travel vendor for roundtrip airfare, 1 night of lodging, and 1 day of meals.

We measured actual costs by tracking invoiced expenses for the 6 focus groups. We calculated actual costs on 3 levels: costs per participant, costs per group with actual participation (ie, actual show rates), and costs per group assuming full participation (ie, 8 participants per group).

#### Recruitment Measures

We measured recruitment in several ways. First, we examined enrollment timing by using the daily recruitment updates to assess how quickly participants were enrolled in each mode. Second, we examined show rates for each focus group mode. Third, we examined participant demographics by mode, including age, sex, race, education, income, employment status, geographic location, urban-rural classification, public transportation use, Internet use, health care utilization, body mass index (BMI), and personal health ratings. We captured this demographic information on the recruitment screener. Most measures were self-reported; however, we calculated BMI using self-reported height and weight, and we identified participants’ urban-rural classifications by comparing their home zone improvement plan (ZIP) codes against the National Center for Health Statistics’ urban-rural classification scheme for counties [22].

#### Participant Logistic Measures

We measured participant logistics using several items from the exit questionnaire. Specifically, we assessed willingness to participate in future groups (“If I were invited to join another [in-person or online] focus group, I would do it.”); perceived difficulty attending (“How easy or difficult was it to join today’s focus group?”); and preparation and travel time (“How much time did you spend preparing for and traveling to today’s focus group?”). The first 2 items had 6-point response scales, and the last item was open ended (measured in minutes).

For virtual group participants, we also assessed participant location during groups (“Where were you during today’s focus group?”); device used by participant (“What type of device did you use to join today’s focus group?”); and individuals in the vicinity (“How many people—besides yourself—were near you during the focus group?”). We calculated percentages and means by mode for the participant logistic measures.

## Results

### Costs (Projected and Actual)

**Figure 4:**
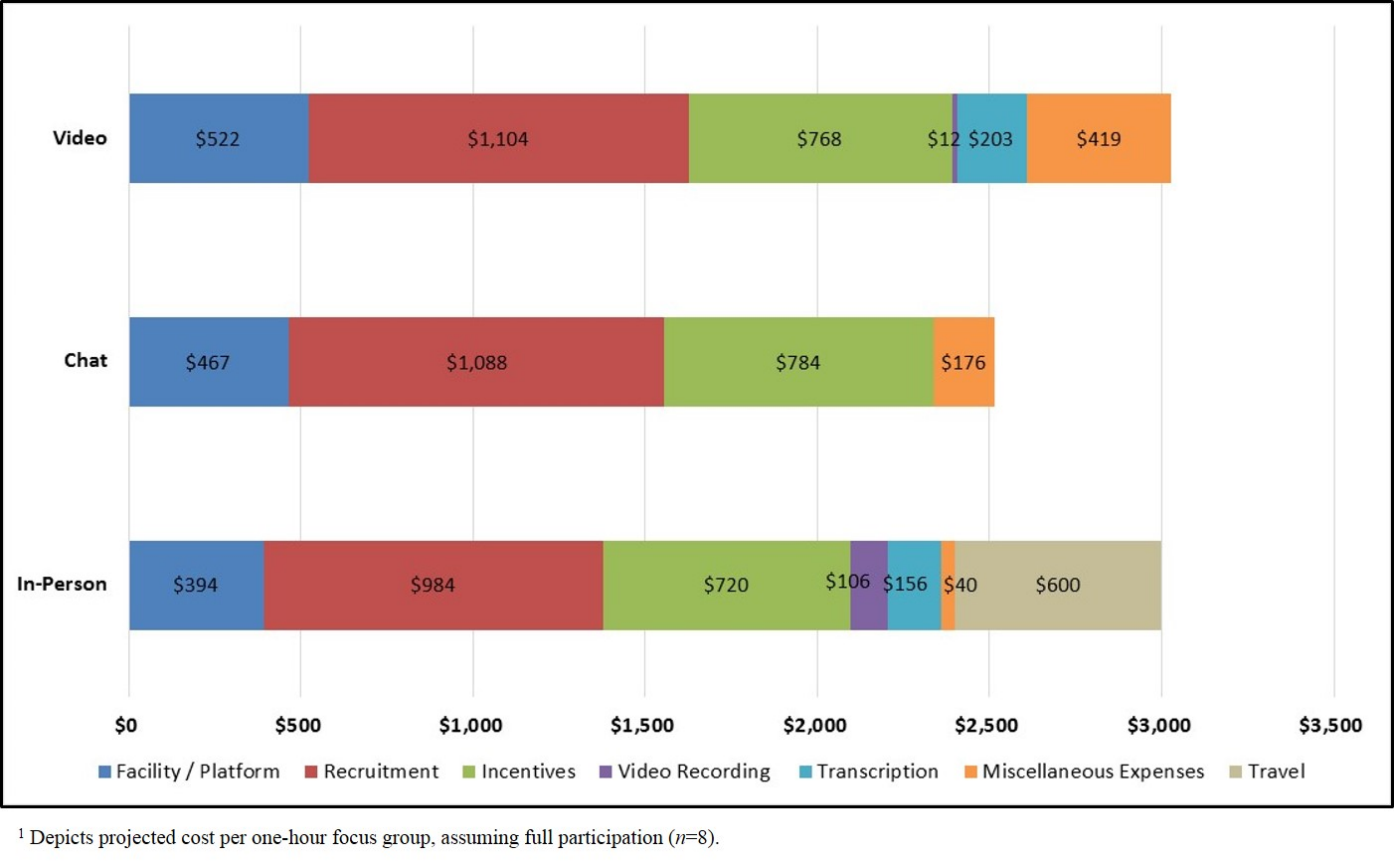
Projected costs by focus group mode.

The differences in projected costs by focus group mode were minimal, with in-person groups projected to cost US $3000 per session compared with US $2515 per session for chat groups and US $3028 per session for video groups (Table 3 and Figure 4). Although video recording and travel costs were notably higher for in-person groups, the platform costs and miscellaneous costs were considerably higher for chat and video groups. Recruitment and recommended incentive costs were nearly identical across modes.

Several competing factors accounted for the minimal differences in price. On one hand, recording costs were minimal for video groups and nonexistent for chat groups, and transcription costs also were nonexistent for chat sessions. Likewise, neither chat nor video groups budgeted for research team travel. On the other hand, platform rental for chat and video groups was slightly more expensive than space rental for in-person focus groups, and virtual groups also projected multiple miscellaneous expenses, such as management fees (US $250 per project), incentive mailing fees (US $5-10 per participant), and Web cameras (US $75 per participant, as needed).

**Table 3:** Projected costs by focus group mode.

Expense category (in US $)^a^	Focus group mode		
	In-person	Chat	Video
Facility or platform	394	467	522
Recruitment	984	1088	1104
Incentives	720	784	768
Video recording	106	N/A^b^	12
Transcription	156	No cost	203
Miscellaneous	40	176	419
Travel	600	No cost	No cost
Total expenses	3000	2515	3028

^a^Depicts projected cost per 1-hour focus group, assuming full participation (n=8).

^b^N/A: not applicable.

**Figure 5:**
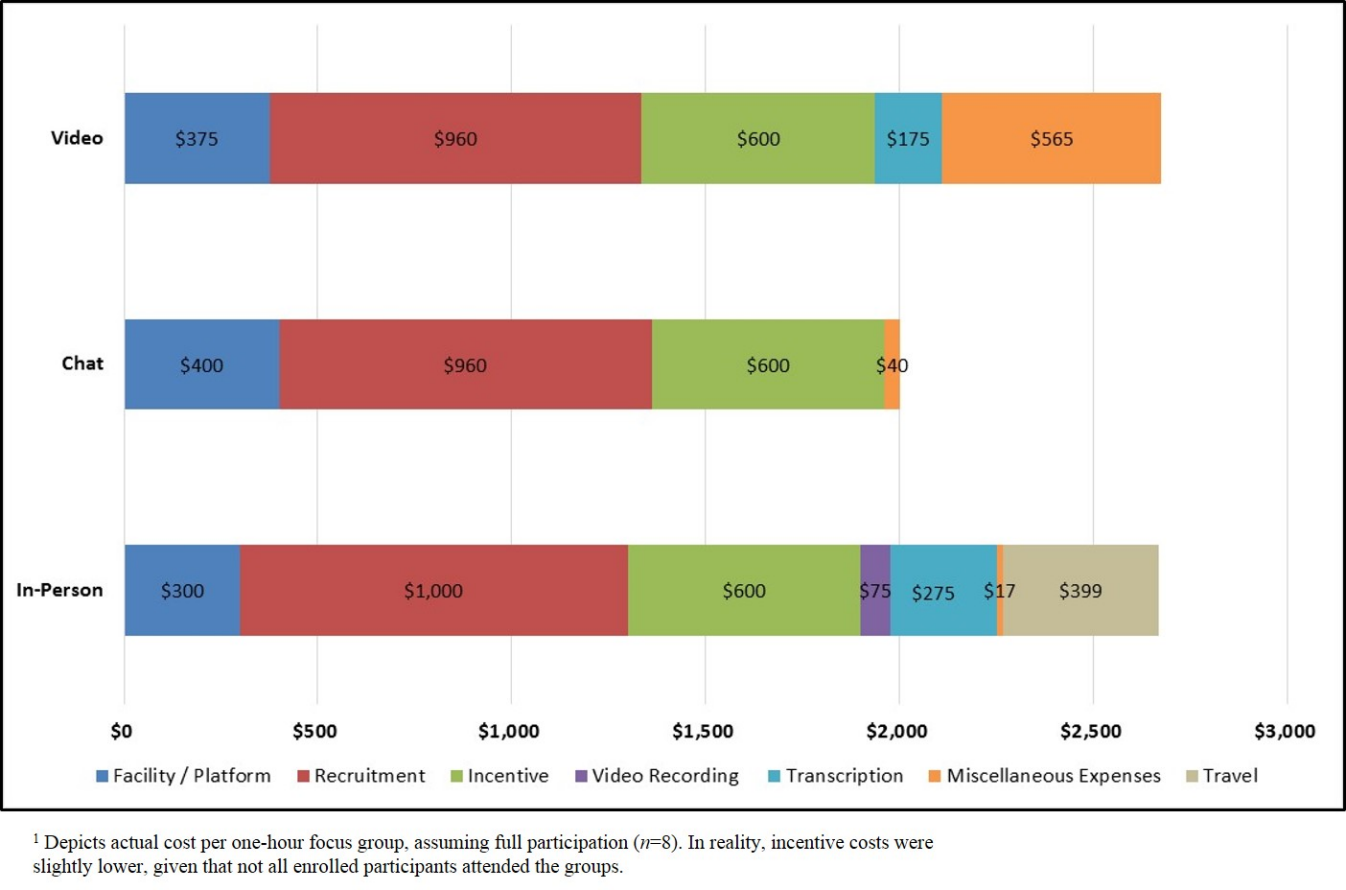
Actual costs by focus group mode.

The differences in actual costs by focus group mode were very similar to the differences in projected costs, with chat groups (US $2000) being less expensive than in-person (US $2666) and video (US $2675) groups (Table 4 and Figure 5). Although in-person groups incurred travel costs and higher recording and transcription fees, virtual groups still had higher platform costs and more miscellaneous expenses (eg, incentive mailing fees, participant Web cameras). Of particular note, 14 of the 16 individuals enrolled in the video groups did not have Web cameras, and the research team needed to purchase cameras for these participants (We standardized actual incentive amounts across the study to ensure a fair comparison on recruitment metrics. Consequently, incentive costs did not differ by mode).

Actual costs for the focus groups in all 3 modes were slightly lower than projected for several reasons. First, the facility or platform costs were lower than anticipated, especially for video groups. Second, we offered slightly lower participant incentives than recommended by the recruitment firms (US $75 vs US $90-98); we selected this incentive based on the amount typically approved for federal government research. Finally, actual travel costs were only two-thirds of projected travel expenses.

**Table 4:** Actual costs by focus group mode.

Expense category (in US $)^a^	Focus group mode		
	In-person	Chat	Video
Facility or platform	300	400	375
Recruitment	1000	960	960
Incentives	600	600	600
Video recording	75	N/A^b^	No cost
Transcription	275	No cost	175
Miscellaneous	17	40	565
Travel	399	No cost	No cost
Total expenses	2666	2000	2675

^a^Depicts actual cost per 1-hour focus group, assuming full participation (n=8). In reality, incentive costs were slightly lower, given that not all enrolled participants attended the groups.

^b^N/A: not applicable.

### Recruitment and Participant Characteristics

#### Enrollment Timing

**Figure 6:**
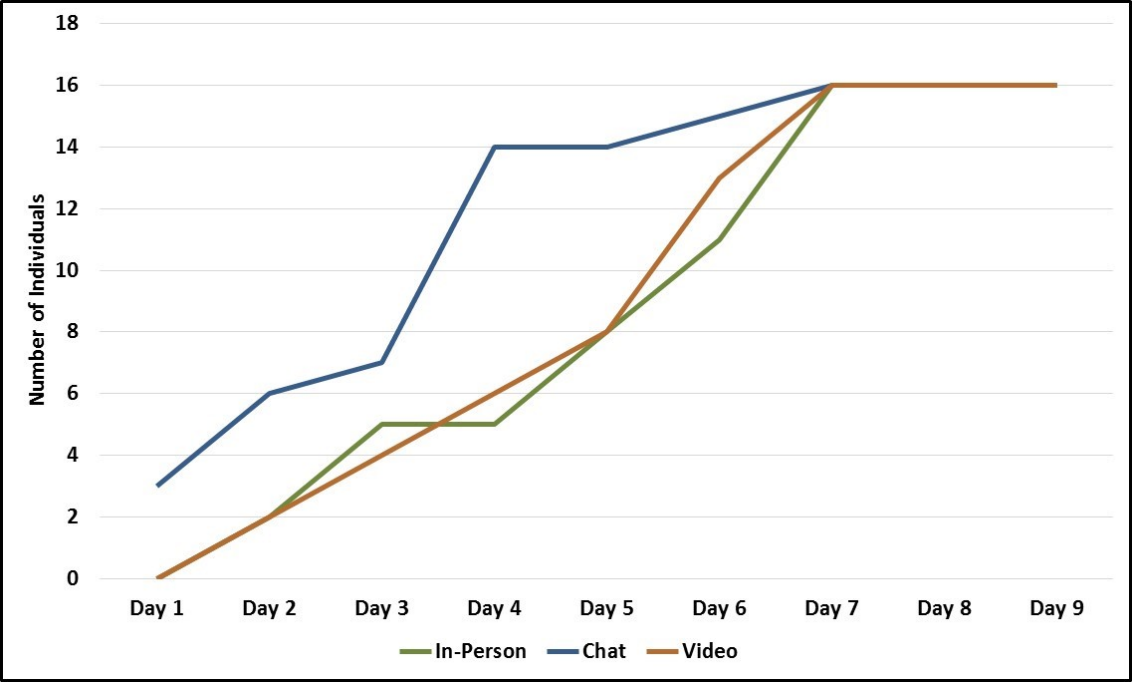
Participant enrollment timing by focus group mode.

Recruiters for all 3 focus group modes were able to complete participant enrollment in 7 days, with enrollment finishing on the same day for all 3 modes (Figure 6). However, chat groups initially enrolled participants more quickly, with in-person and video groups lagging behind until the final day. Conversely, the in-person and video group enrollment rates were nearly identical throughout the recruitment period.

#### Show Rates

**Figure 7:**
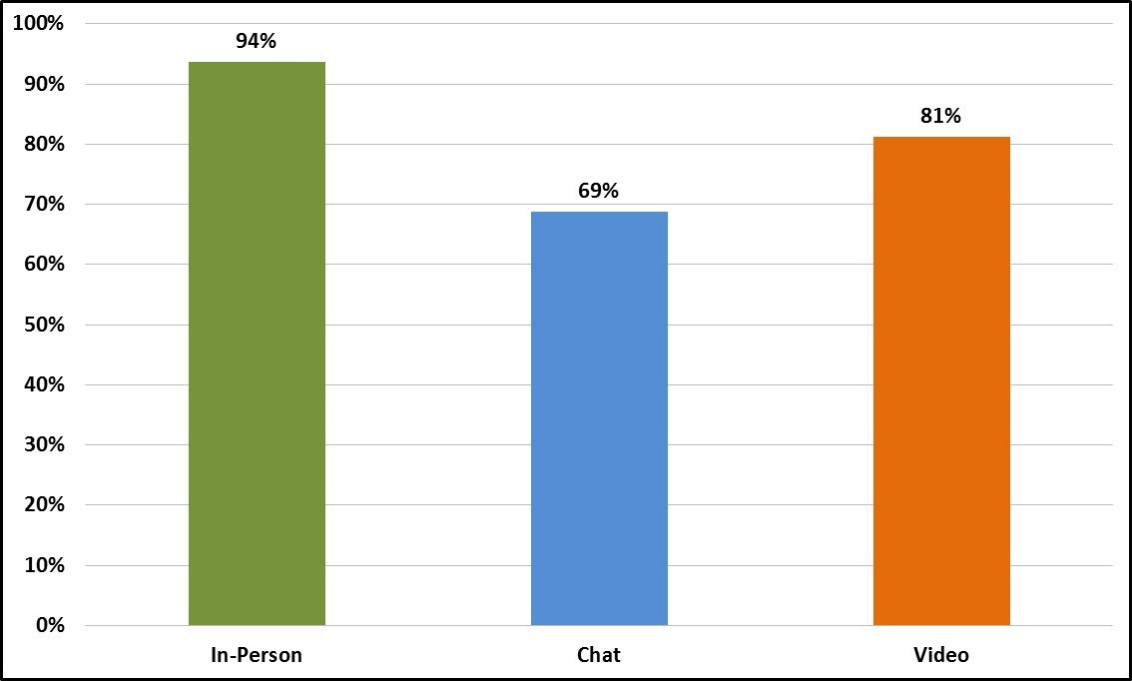
Show rates by focus group mode.

Although all 6 focus groups were fully enrolled (n=8 per group), show rates were noticeably different by group mode (Figure 7). In-person groups had the highest show rates (94%, 15/16), followed by video groups (81%, 13/16) and chat groups (69%, 11/16). Of the enrolled individuals who did not participate, those in the in-person and video groups all cancelled by alerting recruiters a few hours in advance that they would not be able to attend. By contrast, most of the enrolled individuals who did not participate in the chat groups simply did not show up for the sessions or were excluded because they arrived more than 20 minutes late.

#### Geographic Diversity

**Figure 8:**
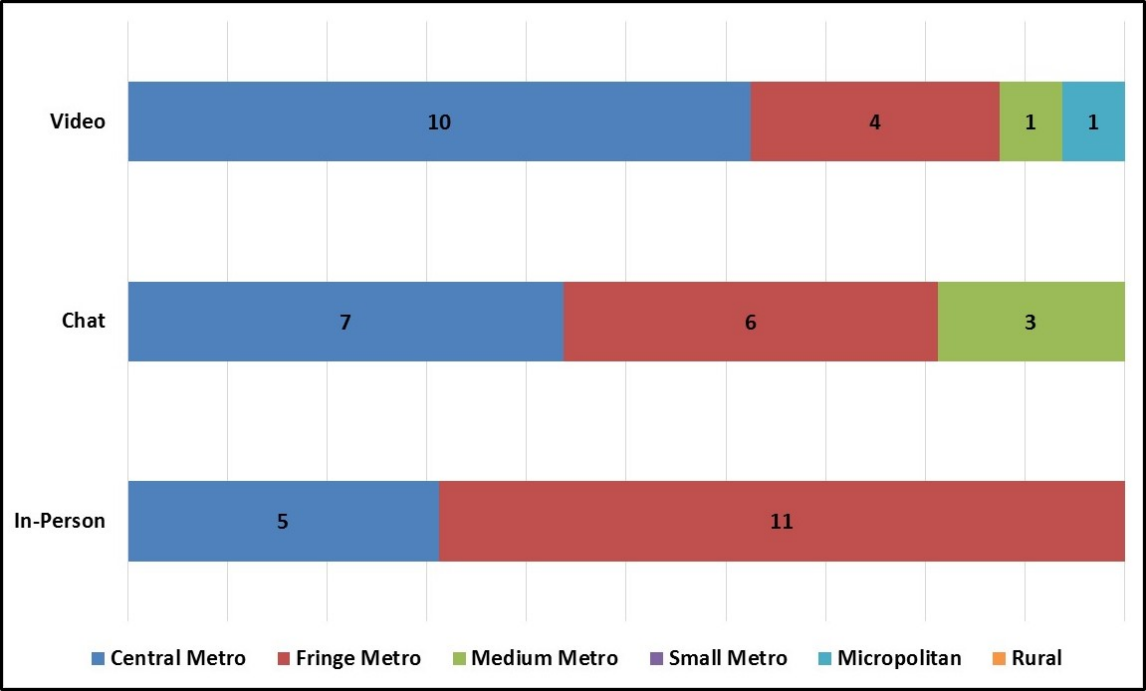
Number of participants per urban-rural classification by focus group mode.

As anticipated, chat and video group enrollees represented a greater number of US states and a slightly broader range of geographic areas in terms of urban-rural classification (Figure 8). Specifically, individuals enrolled in the in-person groups all came from the same state (Georgia) and resided in central and fringe metro areas. In contrast, chat and video groups enrolled individuals from 11 states and 10 states, respectively, and enrolled at least a few individuals who resided in more outlying areas (eg, medium metro, micropolitan). Nevertheless, chat and video groups still recruited heavily from central and fringe metro areas and no focus group mode enrolled individuals from rural areas.

**Figure 9:**
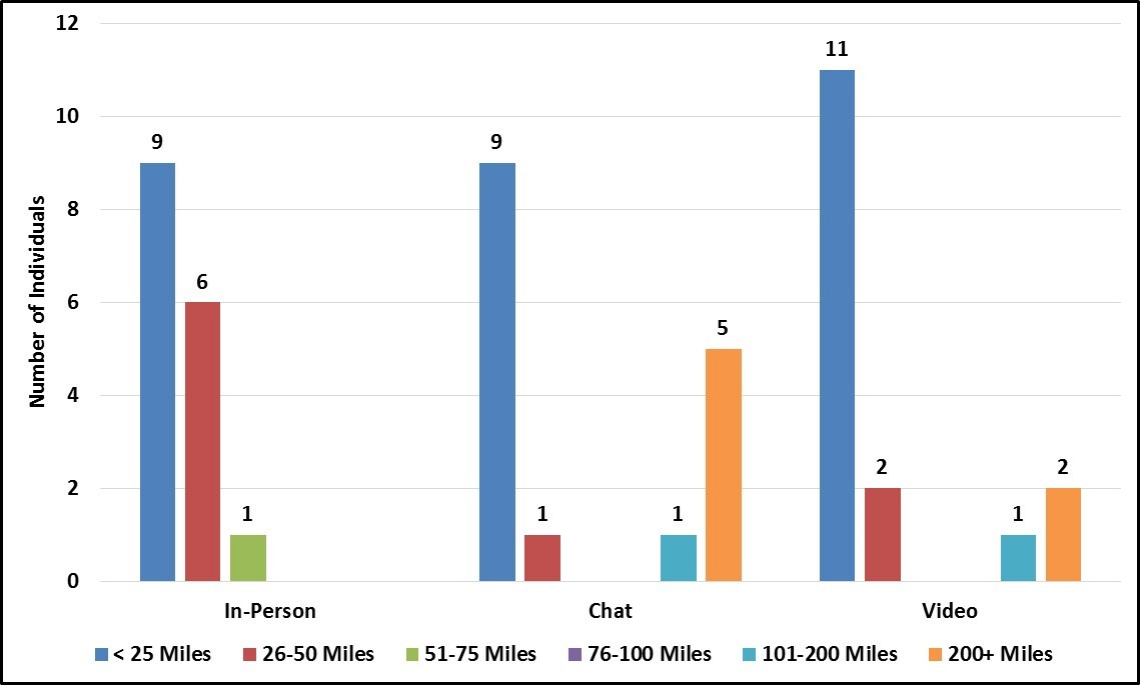
Participant proximity to recruitment firm offices by focus group mode.

We also examined enrolled individuals’ geographic proximity to the recruitment firm’s satellite offices to determine how often virtual group recruiters relied on satellite office participant databases (rather than searching nationally for potential participants). Surprisingly, more than half of the individuals enrolled in the chat (62%, 10/16) and video (81%, 13/16) groups lived within 50 miles of one of the recruitment firm’s 14 satellite offices (Figure 9). Thus, the geographic diversity of the virtual group enrollees was closely tied to the recruitment firm’s physical locations rather than dispersed across the country.

#### Participant Demographics

We examined age, race or ethnicity, education, and income by focus group mode. On average, individuals enrolled in the in-person groups (mean 52.3 years) were slightly older than individuals enrolled in the chat (mean 48.8 years) and video (mean 45.2 years) groups. Moreover, no video group enrollees were older than age 60 years, whereas almost 40% (6/16) of the in-person and chat group enrollees were 61 years or older.

In terms of race, the chat and video group enrollees were more diverse than the in-person group enrollees. The chat and video groups also enrolled individuals from a broader range of racial categories, representing 4-5 different races (white, black, Hispanic, Asian, and other) whereas the in-person groups enrolled only white and black individuals.

Chat and video groups enrolled individuals with a broader range of educational backgrounds than those enrolled in the in-person groups. Although chat groups enrolled the highest number of individuals with post-graduate degrees, the chat and video groups also enrolled a sizeable number of individuals with less than a high school education—25% (4/16) and 38% (6/16), respectively. In contrast, the in-person groups were unable to recruit any individuals without a high school education, despite considerable pressure to do so from the research team.

On average, chat group enrollees had a noticeably higher household income (mean US $71,438) than in-person (mean US $51,750) and video (mean US $49,500) group enrollees. In-person groups also enrolled a greater number of low-income individuals (household income less than US $30,000) than the other 2 two modes. This is surprising, given that in-person groups were unable to recruit individuals with less than a high school education, and it might suggest that in-person groups were more likely to recruit educated individuals who worked low-income jobs or were retired.

#### Internet Use

**Figure 10:**
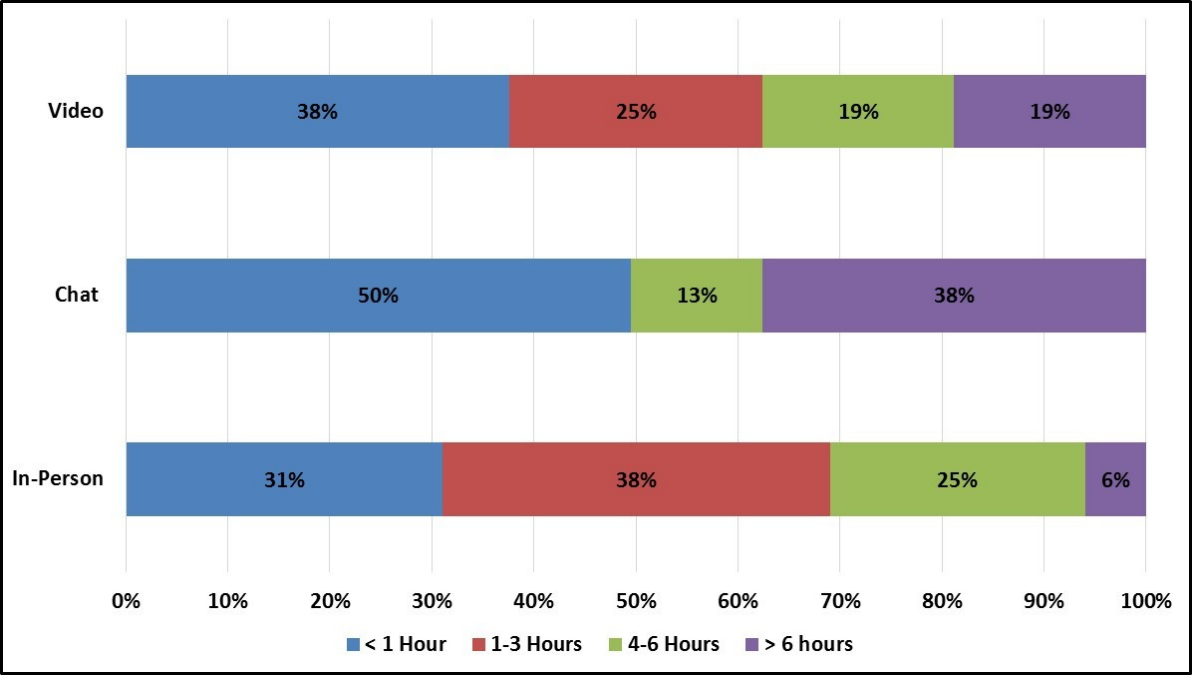
Participant’s Internet use at work by focus group mode.

**Figure 11:**
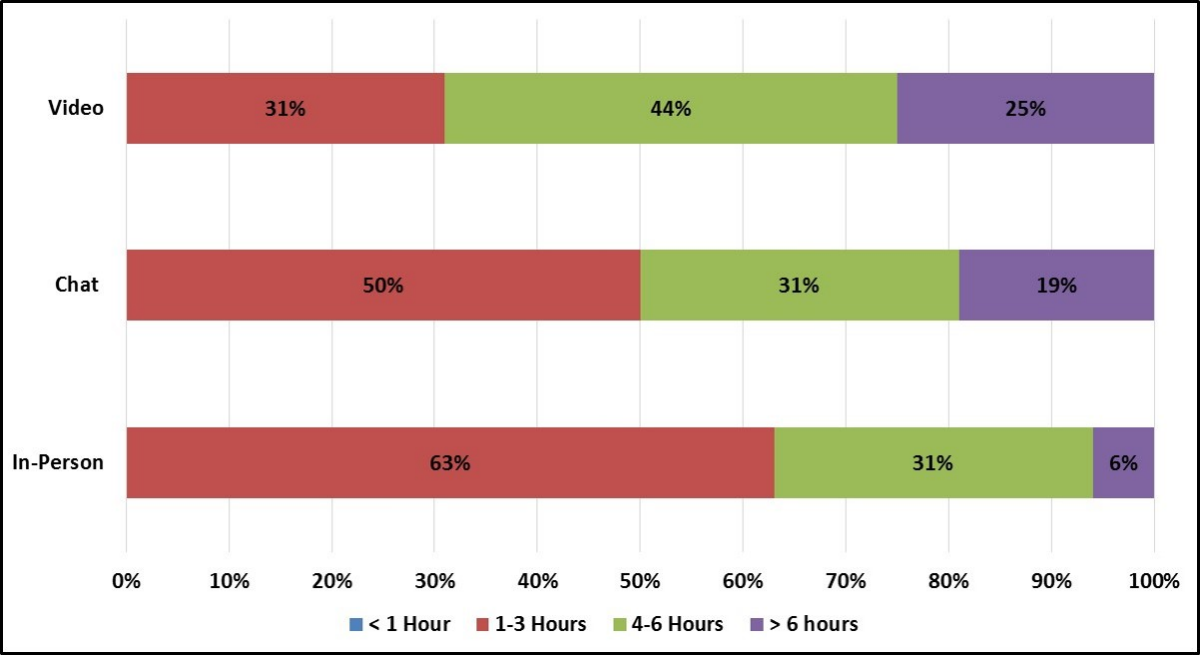
Participant’s Internet use at home by focus group mode.

Given that virtual focus groups require online access, we examined differences in daily Internet use by focus group mode. Not surprisingly, chat and video group enrollees reported higher daily Internet use at both work and home than in-person group enrollees (Figures 10 and 11). However, chat and video groups still enrolled a sizeable number of light Internet users, especially individuals who use the Internet infrequently at work.

#### Health Status and Utilization

**Figure 12:**
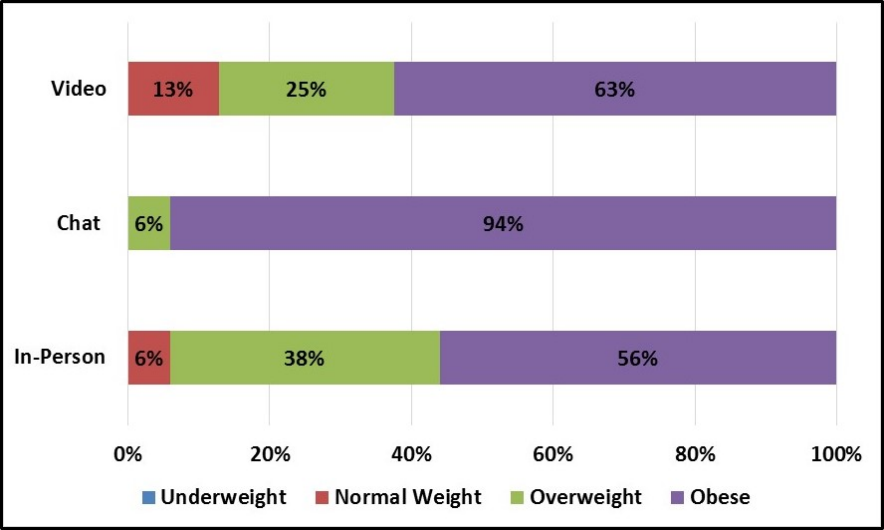
Participant body mass index (BMI) values by focus group mode.

**Figure 13:**
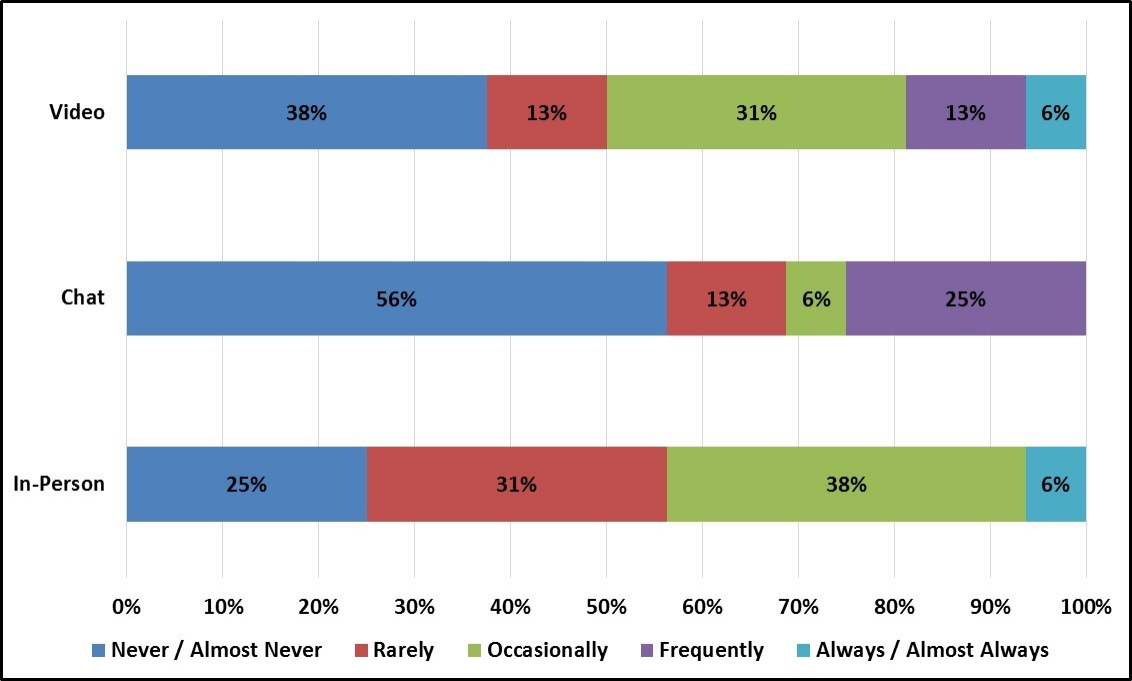
Frequency of participant health interference in relationships by focus group mode.

**Figure 14:**
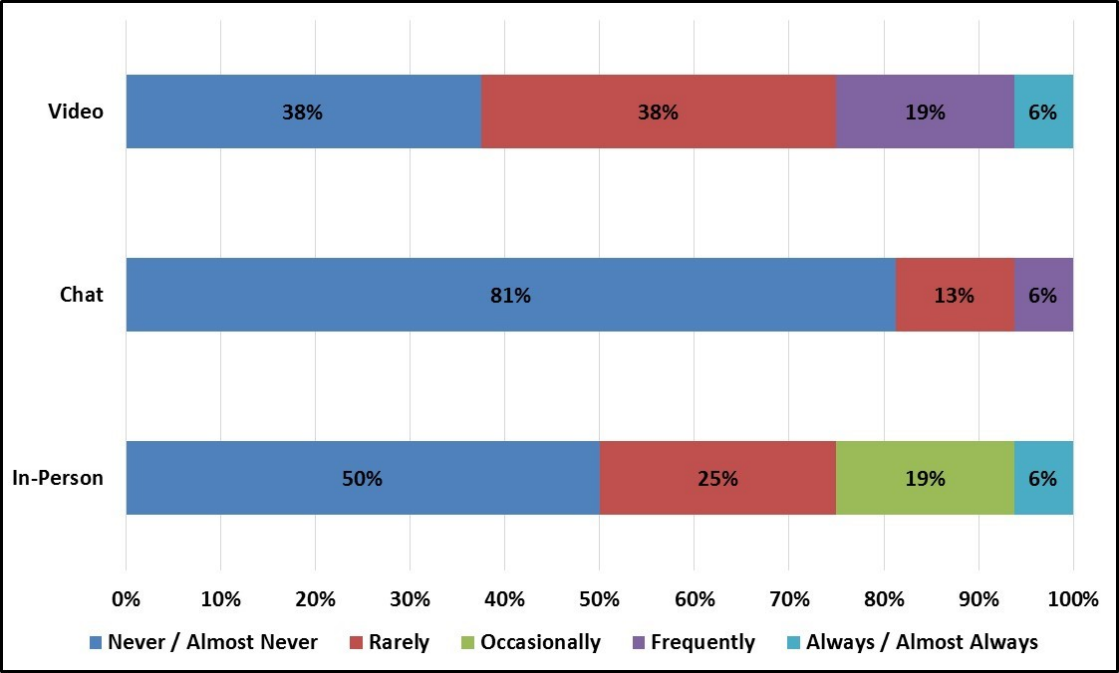
Frequency of participant health interference with job by focus group mode.

Chat and video group enrollees reported being in poorer health than in-person group enrollees on several dimensions, including BMI (Figure 12) and health interference in daily activities (Figures 13 and 14). Chat group enrollees, in particular, reported very high BMI values, with all but 1 categorized as obese. One video group enrollee also reported a BMI value of 65.2, more than double the threshold for obesity. Likewise, chat and video group enrollees were more likely to report that their health interfered with their relationships and, among video group enrollees, their jobs.

In terms of health care utilization, fewer video group enrollees reported visiting a health care provider in the last month compared with enrollees in the other modes. Specifically, only 31% (5/16) of video group enrollees reported visiting a health care provider in the previous month compared with 63% (10/16) of chat enrollees and 75% (12/16) of in-person enrollees. On one hand, this finding may suggest better health among video group enrollees because they use health services less frequently. On the other hand, this finding may indicate that video group enrollees encounter more barriers to accessing health services (eg, no insurance, long distance from facility).

### Participant Logistics

**Figure 15:**
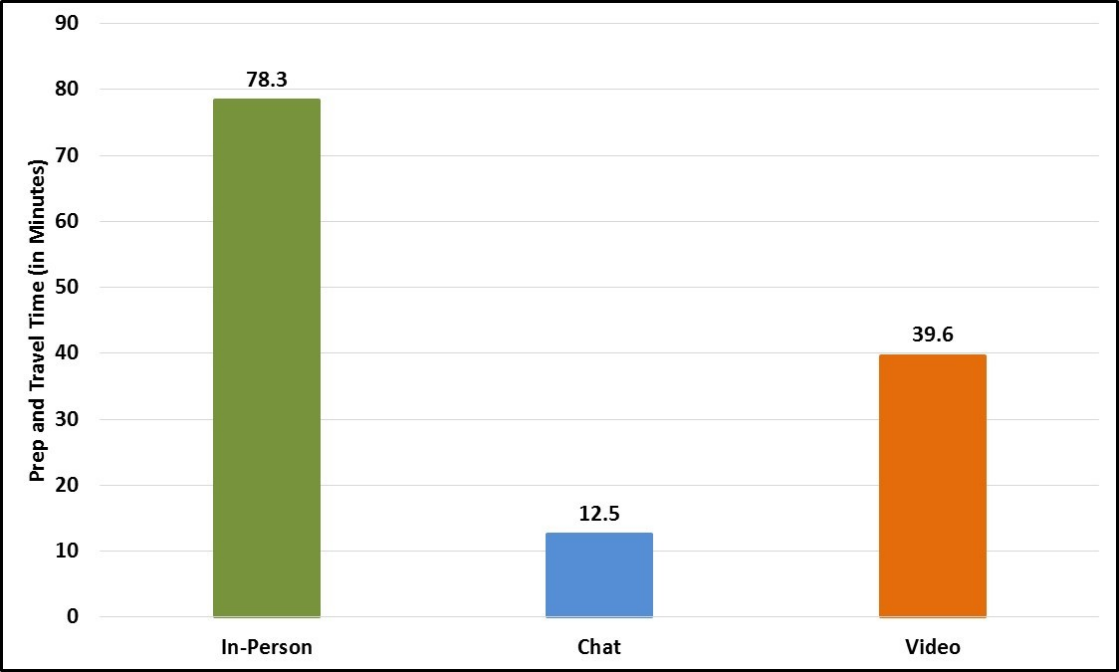
Average participant preparation and travel time by focus group mode.

Overall, individuals who joined in-person focus groups reported a slightly higher participation burden than individuals who joined chat and video groups. Specifically, participants rated in-person groups as slightly more difficult to join (mean rating 2.4 out of 6.0) than chat (mean rating 1.0) and video (mean rating 1.3) groups. Participants also spent noticeably more time preparing for and traveling to in-person groups than they did preparing for virtual groups (Figure 15). Nevertheless, this additional burden was not reflected in participants’ intentions, and participants generally expressed a high willingness to participate in future focus groups using the same mode (in-person = mean rating 5.9 out of 6.0; chat = mean rating 6.0; video = mean rating 5.5).

In terms of virtual focus group logistics, almost all chat and video group participants reported joining the sessions from their homes (91% [10/11] and 100% [13/13], respectively), and all virtual group participants logged onto the platforms using a laptop or desktop computer. No participant joined a group using a tablet, smartphone, or other mobile device. Most participants (71%, 17/24) also joined the virtual groups with no other individuals in their immediate vicinity. However, a handful of participants reporting having 1 or 2 individuals nearby, and 2 chat group participants reported having 5 or more people near them during the sessions.

## Discussion

### Principal Findings

There are a number of hypothetical benefits to conducting virtual focus groups, including reduced expenses, faster turnaround time, increased participant diversity, recruitment of hard-to-reach and low-prevalence populations, and reduced participant burden. However, no study has rigorously compared virtual and in-person focus groups to determine whether these benefits materialize in practice. This study presented the most thorough research to date on virtual focus group costs, recruitment, and logistics, and the findings provided a concrete evidence base for understanding the advantages and limitations of virtual qualitative research. We address each of the proposed benefits of virtual focus groups in the following sections.

#### Claim #1: Virtual Focus Groups Are Less Expensive

We found that both projected and actual cost differences by focus group mode were minimal, with chat groups costing only about US $500- US $600 less and video groups costing approximately the same as in-person groups. Although virtual groups do eliminate the need for travel, they typically incur other costs (eg, management fees, Web camera purchases) that offset this potential savings. Moreover, despite the allegedly decreased burden of participating in a virtual group, recruitment firms often recommended monetary incentives that were higher for virtual groups than for in-person groups.

Show rates are another cost factor that should be considered. We experienced a higher number of cancellations and no-shows among virtual group participants, especially in the chat groups (One possible explanation is that the lack of visual interaction in chat groups leads to a decreased sense of accountability among enrolled individuals). Because cancellations and no-shows still incur recruitment costs and because researchers may need to enroll additional individuals to offset those who withdraw, this reduced participation has cost implications.

#### Claim #2: Virtual Focus Groups Provide Faster Data

Factoring in travel time, chat and video groups clearly require less time to conduct, and they enable researchers to conduct groups in multiple geographic locations within a single day. That said, when excluding travel, we found that research team preparation time for virtual groups was roughly equivalent to preparation time for in-person groups. On one hand, virtual groups might require additional preparation activities that were unnecessary during in-person groups, such as uploading questions into the chat platform, programming electronic consent forms and exit questionnaires, sending confirmation letters, mailing incentives, and addressing unforeseen technology issues (eg, Web camera not working properly).

On the other hand, virtual groups might streamline or eliminate activities that require more time during in-person groups, such as transcripts (instantly available from chat groups), bathroom breaks (no need for participants to ask), and directing lost participants (no facility for participants to locate). Other activities—such as participant enrollment—did not appear to differ by focus group mode.

#### Claim #3: Virtual Focus Groups Increase Participant Diversity

Virtual groups theoretically increase participant diversity by accessing a broader range of individuals, particularly in terms of geography. Our study findings support this claim. Participants in the chat and video groups represented a wider range of geographic areas in terms of both states and urban-rural classification. However, we also found that most virtual group participants lived in close proximity to 1 of the recruitment firm’s satellite offices, suggesting that virtual participant diversity was likely tied to the recruitment firm’s geographic locations. In addition, people residing in rural areas still were not represented in virtual groups. These findings suggest that researchers might need to push recruiters to enroll individuals outside of their established recruitment pools in order to maximize the geographic reach offered by virtual groups.

We also found that virtual groups enrolled more diverse participants in terms of race or ethnicity, education, and household income. In particular, virtual groups were more effective at enrolling individuals with less than a high school education, individuals of Asian and Hispanic background, and individuals with higher household incomes—all groups that could be difficult to reach through focus groups. Conversely, in-person and chat groups were equally adept at enrolling individuals of different ages, but video groups were unable to enroll any individuals older than 60 years.

One hypothetical limitation was that virtual focus groups might enroll primarily individuals who were frequent Internet users and who were adept at online technology. Although daily Internet use was generally higher among virtual group participants than among in-person group participants, virtual groups still enrolled a sizeable number of light Internet users who spent less than 1 hour per day on the Internet at work. This might suggest that virtual groups were able to enroll individuals who worked in blue collar, retail, and service fields and spent little time working at a computer.

#### Claim #4: Virtual Focus Groups Capture Hard-to-reach Populations

Given that we intentionally recruited individuals with diabetes for this study, we did not assess whether virtual focus groups enrolled individuals with low-prevalence health conditions. Nevertheless, we did find that virtual groups enrolled less healthy individuals than in-person groups, as evidenced by the number of participants with extremely high BMIs (extremely obese) and the number of individuals who reported that their health interfered with their relationships. As mentioned previously, virtual focus groups also enrolled more individuals from hard-to-reach demographic groups, such as those with a high school education or less, those with high family incomes, and those of a race other than black or white.

#### Claim #5: Virtual Focus Groups Reduce Participant Burden

We found that chat and video groups slightly reduced the logistical burden on participants compared with in-person focus groups. On average, virtual group participants spent noticeably less time preparing for the sessions, and they rated the groups as easier to join. However, virtual groups did have higher cancellation and no-show rates, and there was no meaningful difference by mode in participants’ willingness to join another focus group using the same methodology. These findings suggest that, although virtual focus groups reduced participant burden, this feature did not necessarily translate into more active or engaged participants.

### Limitations

This study offered several advantages over previous research on virtual focus groups, including a direct comparison of 3 modes, objective measurements, and strong experimental controls. Nevertheless, this study does have limitations that need to be considered when interpreting the findings. First, the study had a modest sample size, in terms of both groups and participants, which increases the likelihood that a few data points might have skewed the findings. Second, we conducted the study with a single illness population (type 2 diabetes), which might restrict the generalizability of the findings. Although we selected this population because of its demographic diversity, it is possible that replicating this study with other populations would lead to different results.

Third, we used a single recruitment firm to identify and enroll participants, which means that some results might be influenced by the firm’s procedures and infrastructure. We selected a single firm to standardize recruitment across modes, and we blinded recruiters to the other modes to ensure impartiality. Although we believe these experimental controls outweigh the potential for firm bias, it is a possible limitation. Finally, we encountered a video group scheduling error (ie, second video group scheduled at 7:00-8:00 PM Eastern Daylight Time instead of 8:00-9:00 PM Eastern Daylight Time) that inferred with the study’s experimental time control. All participants were aware of the group starting time. Therefore, it is unlikely that this error affected show rates; however, the earlier timeslot might have affected participant availability.

### Future Research

This study is an important and rigorous step in establishing the evidence base on virtual focus groups. However, we recommend additional research in this area to broaden and deepen our understanding of this methodology. First, future research should replicate and expand this study with more focus groups, more participants, and more timeslots to see whether the results change. Second, future studies should examine additional populations beyond type 2 diabetes, including hard-to-reach groups (eg, health care providers, individuals with physical disabilities) and sensitive illness populations (eg, sexually transmitted illnesses, clinical depression). Finally, this study addressed only the cost, recruitment, and logistical aspects of virtual focus groups, and future studies need to compare virtual and in-person groups in terms of data quality (eg, group dynamics, data breath, data saturation). This final piece is particularly important, as the recruitment and logistical advantages of virtual focus groups may carry less weight if these groups produce inferior data.

### Conclusions

**Figure 16:**
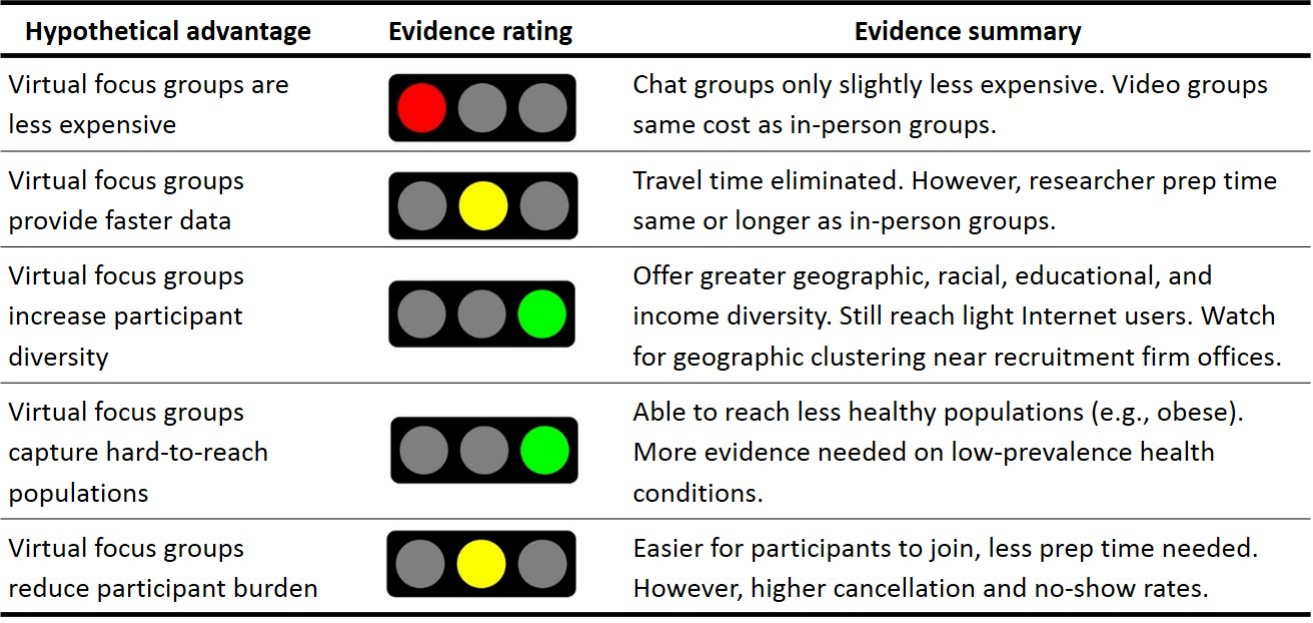
Summary of hypothetical advantages and supporting evidence for virtual focus groups.

Virtual focus groups are an appealing and reasonable option for health and medical researchers who seek faster data, increased participant diversity, and inclusion of hard-to-reach populations in qualitative research (Figure 16). Although virtual groups are unlikely to save money, they do appear to reach a broader range of individuals in terms of geography, race or ethnicity, education, and income, and they seem to offer an advantage when it comes to reaching less healthy individuals and those with mobility impairments. Virtual focus groups also reduce the burden on participants by eliminating travel and minimizing preparation time; however, these advantages do not translate into more active participation, and virtual groups are likely to have higher cancellation and no-show rates. Future research on virtual focus groups should examine data quality to determine whether the methodology’s recruitment and logistical advantages lead to useful data.

## Abbreviations

BMI: body mass index
